# Thermophysical Profile of Industrial Graphene Water-Based Nanofluids

**DOI:** 10.3390/nano14171401

**Published:** 2024-08-28

**Authors:** Soulayma Gal, David Cabaleiro, Walid Hassen, Anaghim Nasri, Yannick Lafue, Cuong Pham-Huu, Housseinou Ba, Patrice Estellé

**Affiliations:** 1 LGCGM, University Rennes, 35000 Rennes, France; soulayma.gal@univ-rennes.fr; 2 LMES, Université de Monastir, Monastir 5000, Tunisia; hassen.walid@gmail.com; 3 CINBIO, Universidad de Vigo, 36310 Vigo, Spain; dacabaleiro@uvigo.gal; 4 BLACKLEAF SAS, 67400 Illkirch-Graffenstaden, France; anasri@blackleaf.fr (A.N.); ylafue@blackleaf.fr (Y.L.); hba@blackleaf.fr (H.B.); 5 ICPPEES, Université de Strasbourg et Centre National de Recherche Scientifique, 67000 Strasbourg, France; cuong.pham-huu@unistra.fr

**Keywords:** few-layer graphene, industrial nanofluids, thermophysical properties, concentration influence, theoretical predictions

## Abstract

The exceptional properties of high-grade graphene make it an ideal candidate for thermal dissipation and heat exchange in energy applications and nanofluid development. Here, we present a comprehensive study of few-layer graphene (FLG) nanofluids prepared in an industrial context. FLG nanofluids were synthesized through an ultrasound-assisted mechanical exfoliation process of graphite in water with a green solvent. This method produces FLG of high structural quality and stable nanofluids, as demonstrated by electron microscope, dynamic light scattering and ζeta potential analyses. Thermal conductivity measurements of FLG-based nanofluids were conducted in the temperature range of 283.15 K to 313.15 K, with FLG concentrations ranging from 0.005 to 0.200% in wt. The thermal conductivity of FLG nanofluids is up to 20% higher than water. The modeling of nanofluid thermal conductivity reveals that this enhancement is supported by the influence of the thermal resistance at the FLG interface, and the content, average dimensions and flatness of FLG sheets; this latter varying with the FLG concentration in the nanofluid. Additionally, the density and heat capacity of FLG suspensions were measured and compared with theoretical models, and the rheological behavior of FLG nanofluids was evaluated. This behavior is mainly Newtonian, with a weak 5% viscosity increase.

## 1. Introduction

The transition to a climate-neutral society is a challenging and great opportunity within the energy sector. Efficient transfer and thermal energy storage for sustainable and economic development in order to cover energy production and use needs is one of the key challenges of this century. Enhancing the thermophysical features of usual heat transfer fluids (HTFs), particularly their thermal conductivity, is one of the common strategies for heat transfer process intensification. This can be achieved by the addition of solid particles to nanometer dimension(s), the resulting HTFs being now well-defined as nanofluids. Among the available nature of nanostructures, and compared to usual metal oxides and metal nanoparticles, carbon-based nanostructures have attracted high interest because they exhibit much higher thermal conductivities [1].

Among the carbon nanostructures, graphene stands out. It was discovered by Geim and Novoselov [2,3] and is composed of a single layer of carbon atoms arranged in a sp^2^ configuration. Graphene exhibits unique two-dimensional characteristics, such as high thermal and electrical conductivity. Monolayer graphene is preferred due to its superior attributes compared to multilayer graphene, which can experience reduced thermal conductivity due to interactions between the layers [4]. For this reason, most relevant graphene suspensions contain few-layer graphene (FLG) or graphene nanoplatelets (GNP). FLG generally refers to graphene stacks with 2 to about 5 layers, while graphene layers exceeding 5 and up to 10 (sometimes up to 30) are referred to as multilayer/thick graphene (MLG) [5]. Overall, FLG has remarkable properties, including extreme resistance, transparency, density and excellent electrical and heat conductivity. These properties have sparked extensive research and opened up promising possibilities for various applications [6], including nanofluid usage for heat transfer improvements [7,8].

Thus, graphene-based nanofluids have been extensively studied for their exceptional thermal properties, which exhibit significantly higher thermal conductivities than other nanomaterials, as highlighted in recent state-of-the-art studies [7,9]. Additionally, several studies further confirm the efficacy of graphene-based nanofluids as highly efficient heat transfer fluids (HTFs). Mehrali et al. studied the synthesis of highly water-soluble GNPs through covalent functionalization of GNP sheets with the diazonium salt (DS) of sodium 4-amino azobenzene-4-sulfonate [10]. The authors proved the efficient stability of this nanofluid, which showed maximum sedimentation of 16% after 480 h and stability until 840 h. By incorporating surface-modified GNP sheets into the base fluid, the nanofluid exhibited a thermal conductivity increase of up to 17%. Kumar et al. recently presented a new method for preparing environmentally friendly, non-corrosive and covalently functionalized graphene with gallic acid (GGNP) nanoplatelets as HTF in a liquid planar solar collector to enhance energy performance [11]. Long-term stable and dispersible GGNP water-based nanofluids were prepared at different concentrations of 0.025%, 0.05% and 0.1%. The maximum improvement in liquid planar solar collector efficiency was observed with a 0.1 wt.% GGNP concentration and a flow rate of 1.5 L/min, resulting in a 24.09% increase compared to distilled water. The energy performance analysis revealed that the energy efficiency decreases while the mass flow rate grows, while an increase in nanofluid concentration leads to an improvement in energy efficiency. Lal et al. [12] conducted a detailed study on the convective heat transport performance of f-GnP (functionalized graphene nanoplatelets)-based nanofluids dispersed in distilled water. Their findings demonstrate that functionalized GNP provides better heat transport performance than water as a base fluid. The 0.200% functionalized GNP remains stable for a long period. Additionally, the heat sink temperature decreases by 10 K and both the convective heat transfer coefficient and Nusselt number improve by 10%. The viscosity increases by 13.3% with the addition of GNP. Experimental work was conducted by Cai et al. [13] to investigate the heat transfer performance of a pulsed heat pipe (PHP) employing a hybrid operation mode. The chosen heat transfer fluid was a stable mixture of high-quality FLG multilayer graphene dispersed in a base of water and 40 vol.% ethanol. The study showed that the thermal performance of the PHP with FLG graphene nanofluids was the best (indicating a 25.16% enhancement compared to using water alone) and that Triton X-100 outperformed other surfactants. Recently, Alawi et al. [14] introduced a novel approach for synthesizing covalently functionalized FLG through thermal treatment. The study also investigated the temporal stability and thermophysical characteristics of water-based nanofluids with varying content of functionalized FLG, from 0.025% to 0.1 wt.%. In this last work, the highest nanoparticle concentration of 0.1 wt.% demonstrated an impressive 31% enhancement in thermal conductivity, particularly at 323.15 K.

While many relevant studies have been performed at the laboratory scale, the development of nanofluid technology now demands a particular focus on the industrial production of stable nanofluids, along with an examination of their thermophysical properties for their future usage in real application. As a contribution to this crucial step toward a wider usage of nanofluids, and for the first time in the literature, we report here the complete characterization of FLG nanofluids prepared by a company in an industrial context from mechanical exfoliation of graphite in water under ultrasound and using a green solvent. This method yields high-quality graphene nanoplatelets with a few layers. The structural and morphological aspects of the FLGs were investigated with characterization devices. The stability of the resulting nanofluids was formally assessed using dynamic light scattering (DLS) and zeta potential measurements. Additionally, we measured and analyzed the thermal conductivity of FLG-based nanofluids, considering the influence of temperature (283.15–313.15 K) and graphene concentration (0.005, 0.010, 0.020, 0.050, 0.100, 0.200% in mass). Furthermore, the rheological nature, density and specific heat capacity of the nanofluids were also experimentally evaluated to provide comprehensive knowledge of their thermophysical features. The findings will contribute to the existing research of graphene-based nanofluids, particularly due to the industrial nature of these suspensions, and should have significant implications for future applications in the advanced thermal sector.

## 2. Mateirials and Methods

### 2.1. Nanofluids Preparation from Enhanced Liquid-Phase Exfoliation

Graphene nanofluids were produced by the Blackleaf Company (Illkirch-Graffenstaden, France) using a refined liquid-phase exfoliation approach under a patented process [15]. For the process, Imerys (Tokyo, Japan) supplied high-purity graphite was chosen as the primary feedstock. Simultaneously, karaya gum surfactant, from Thermo Scientific Chemicals (Waltham, MA, USA) was incorporated. This surfactant is recognized for its dual capabilities as an emulsifier and dispersion stabilizer. In accordance with the protocol, an exact weight of 1.0 g of graphite was intimately mixed with a 0.1 g allotment of the designated surfactant within a precision volumetric flask, accommodating 1000 mL of doubly-distilled water, ensuring a regulated environment. This carefully prepared mixture was subjected to ultrasonication using an 80-watt probe sonicator SFX550 Sonifier (Branson, MO, USA). During this phase, continuous mechanical stirring was applied for a consistent period of 2 h to guarantee complete graphite exfoliation. Following successful exfoliation, subsequent meticulous dilutions of the primary solutions were performed, ensuring suitability for the impending series of characterization evaluations. As a result, six different samples were prepared with graphene content ranging from 0.200 to 0.005% in mass.

### 2.2. Characterization Methods

The methods employed for characterizing the graphene sheets, stability and thermophysical characteristics of graphene-based nanofluids are outlined below.

Scanning Electron Microscopy (SEM) analyses were conducted with JEOL 2600F (Chiba, Japan) equipment. An acceleration voltage of 15 kV was applied with the emission current maintained at a constant 10 mA. In order to prevent charging effects during electron microscopy examinations, the specimens were sputter-coated with a thin layer of gold.

Transmission Electron Microscopy (TEM) characterizations were performed using a JEOL 2100F (Japan) device. The microscope was equipped with a probe corrector tailored for spherical aberration corrections, ensuring a precise point-to-point resolution of 0.2 nm. The microscopy has been conducted at an acceleration voltage of 200 kV. To achieve optimal dispersion, the samples were subjected to mild ultrasonication in ethanol for a 5-min duration. Subsequently, aliquots of this dispersion were allocated onto holey carbon TEM grids, facilitating detailed morphological investigations.

Data from Raman Spectroscopy were collected using the Horiba LabRAM ARAMIS system (LabSpec 6 Spectroscopy Suite Software). Measurements were taken over a wavenumber domain ranging from 500 to 4000 cm^−1^, using a 532 nm laser for excitation. To optimize spectral data collection, analytes were applied to pristine glass substrates through a spin-coating method. These substrates were then subjected to drying protocols, ensuring minimal interference during spectral acquisition. All of these characterizations were performed under ambient conditions.

The hydrodynamic size distribution and ζeta potential of graphene nanofluids were investigated at 298.15 K using a Zetasizer Nano ZS device (Malvern Instruments Ltd., Malvern, UK) equipped with a Helium-Neon laser (4 mW, 632.8 nm). The size of dispersed particles was estimated by the Dynamic Light Scattering (DLS) principle with a scattered angle of 173°. ζeta potential evaluations were performed in single-use folded capillary cells using the electrophoretic light scattering technique with a detection angle of 13°. Before ζeta potential analyses, samples were diluted 100 times in Milli-Q water to ensure that the recorded signal fell within the sensitivity range. All measurements were taken in triplicate to ensure representative results. An uncertainty of 5% was considered for size determinations using this technique.

Densities, *ρ*, were obtained using an oscillating U-tube densimeter (DMA 501, Anton Paar, Graz, Austria). The experimental tests were conducted from 288.15 K to 313.15 K. The temperature was tightly controlled with an accuracy of ±0.03 K (repeatability of ±0.01 K) using a solid-state thermostat integrated into the measuring cell. Toluene was selected as the reference material to calibrate the instrument. The density values, *ρ*, obtained with this instrument have an estimated uncertainty of 1 kg·m^−3^.

Isobaric heat capacities, *Cp*, were measured at temperatures ranging from 283.15 K to 353.15 K using a heat-flow-type differential scanning calorimeter, DSC-Q2000 (TA Instruments, New Castle, DE, USA), equipped with a refrigerated cooling unit, RSC90 (TA Instruments, USA). Experiments were conducted from a quasi-isothermal Temperature-Modulated Differential Scanning Calorimetry (TMDSC) method. Samples, weighing (12 ± 1 mg), were hermetically sealed in Tzero aluminum pans and placed in the DSC chamber under a nitrogen atmosphere (purge flux: 50 mL·min^−1^). After stabilizing the tested material at the desired temperature, the DSC heat flow was calibrated to zero. Then, the sample temperature was sinusoidally modulated with an amplitude of 0.5 K and a period of 80 s for about 30 min. *Cp* values were obtained from MDSC reversing heat capacity signal, which had been previously calibrated using water, toluene, ethylene glycol and sapphire. An experimental uncertainty of 3% was estimated for isobaric heat capacity and ±0.3 K (with a repeatability of ±0.1 K) for temperature measurements [16].

Thermal conductivity, *k*, of nanofluids were measured across temperatures ranging from 283.15 K to 313.15 K using the THW-L2 instrument (Thermtest Inc., Hanwell, NB, Canada). This instrument employs the transient short hot-wire technique, conforming to the ASTM D7896 standard, with 2% reproducibility. For the measurements, the wire probe was immersed vertically in a sample holder, which contained around 20 mL of the nanofluid, and then placed in a dry bath. This setup allowed for precise regulation and control of the nanofluid’s temperature at +/−0.5 K. Once the nanofluid reached the desired temperature, a 135-mV power supply was applied to the sample with a measurement duration of 1.5 s to avoid any convection perturbation. This experimental methodology aligns with practices previously established in [8], with further details available in that reference. The probe was previously calibrated with DW, and an average absolute deviation of 1.8% was reported for DW in the studied temperature range. It is worth noting that each reported value of thermal conductivity represents the average of at least 10 replicated tests.

The dynamic viscosity, *µ*, and shear flow behavior of nanofluids were evaluated using a Malvern Kinexus Pro rheometer (Malvern Instruments) with a cone-plane fixture (diameter 60 mm; cone angle 1°) from 283.15 K to 313.15 K. This evaluation is performed as previously described in [17]. Briefly, once the sample had equilibrated for 5 min at the required temperature between the geometries, it was subjected to steady-state shearing by applying a logarithmic ramp in shear stress. An experimental maximum deviation of 4% was experimentally estimated with DW in the tested temperature range. Rheological tests were performed in triplicate.

## 3. Results and Discussion

### 3.1. Structural Properties of Graphene Sheets

**Figure 1:**
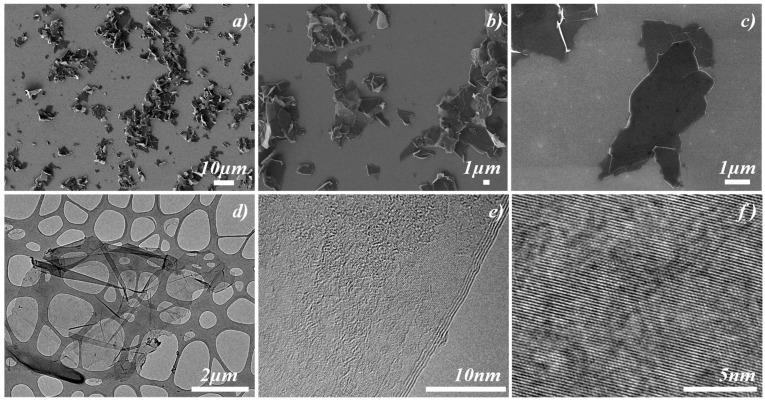
Morphological analysis of FLG sheets obtained via dip-sonication using karaya gum as an exfoliating agent. (**a**–**c**) SEM images; (**d**–**f**) high-resolution TEM (HRTEM) images.

The morphology of the synthesized FLG is comprehensively analyzed using SEM. Figure 1 distinctly shows two-dimensional graphene flake structures with a predominant lateral size centered around 5 μm, as further detailed in Figure 1a–c. From the TEM analyses reported in Figure 1d, it becomes evident that the FLG lateral size is well-centered on 5 nm. Furthermore, these sheets are discernibly constituted by fewer than 10 graphene layers (see Figure 1e). Such observations affirm the heightened level of exfoliation and underscore the efficacy of the adopted exfoliation synthesis protocol. The high-resolution TEM (HRTEM) pictures further elucidate the crystalline nature of the FLG, portraying its well-graphitized surface and the absence of any structural defects, as evidenced by Figure 1f.

**Figure 2:**
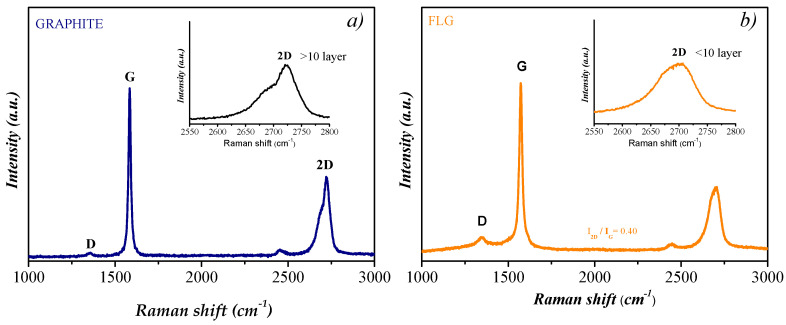
Raman spectroscopic analyses under 532 nm excitation. (**a**) Spectrum of the initial graphite precursor. (**b**) Spectrum of FLG micro-flakes.

To elucidate the structural characteristics and inherent quality of the synthesized graphene, we employed the Raman spectroscopy analytical process. The spectra derived from FLG distinctly highlight the canonical D and G bands, registering peak positions at 1345 cm^−1^ and 1590 cm^−1^, respectively, as illustrated in Figure 2. As corroborated by prior studies [18,19], the D band is indicative of disordered sp^3^ hybridized carbon, while the G band reflects the crystalline properties typical of graphitic sp^2^ carbon configurations. The notably low I_D_/I_G_ intensity ratio lends credence to the heightened graphitization quality of the newly exfoliated FLG. The emergence of the D band at 1376 cm^−1^ can be attributed to potential edge disturbances in the graphene sheets or possible defects on the basal plane, which might arise during the exfoliation. The reduced prominence of the D band across the analyzed samples underscores the efficacy of the adopted exfoliation methodology, adeptly minimizing the induction of structural anomalies [20]. The 2D Raman peak discerned around 2690 cm^−1^, serving as a marker for the layering in graphene, highlights the dominance of structures with fewer than 10 graphene layers, particularly when benchmarked against bulk graphite, as portrayed in Figure 2a.

### 3.2. Stability Analyses

**Figure 3:**
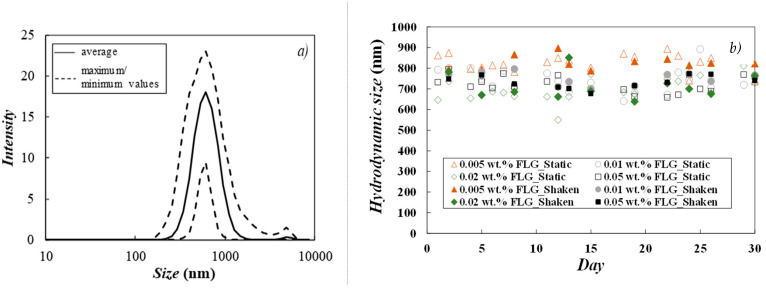
(**a**) Average DLS distributions taken over the 30-day period for 0.050 wt.% FLG nanofluid kept under static conditions and (**b**) changes in particle size for 0.005–0.050 wt.% FLG dispersions over time.

Dynamic Light Scattering (DLS) is a smooth and quick analysis commonly used to assess the hydrodynamic diameter of dispersed particles in different types of colloidal suspensions [21]. The instability of nanofluids may cause nanoparticles to cluster together and eventually sediment. Thus, an increase in apparent DLS diameter with time can be an indicator of sample instability due to nanoparticle agglomeration or aggregation. In this work, dynamic light scattering analyses were repeated for about 30 days to monitor possible changes in the hydrodynamic size of dispersed particles [22]. Only 0.005, 0.010, 0.020 and 0.050 wt.% nanofluids were investigated using this technique, as 0.100 wt.% and 0.200 wt.% FLG loadings were too concentrated to ensure appropriate optical properties. For each concentration, two different samples were studied. One was kept static, and the other was manually shaken for a few seconds just before measurements [23], without any specific visual sedimentation observed. As an example, Figure 3a presents the fluctuations in the particle size distribution of a representative sample (0.050 wt.% FLG nanofluid maintained under static conditions). Dispersions present two-peak DLS distributions with a main peak at about ~800 nm and a secondary low peak at ~5 μm. It should be stressed that dynamic light scattering is based on the assumption that disperse materials are spherical while graphene is sheet-like shaped with only one dimension in the nanometric range. The average DLS sizes, with time, in static and shaken dispersions, are shown in Figure 3b. In the investigated samples, the hydrodynamic sizes of dispersed particles are within 600 to 900 nm, with an average value of 750 ± 50 nm in the whole investigated period.

ζeta potential is a measure of the electrical repulsion that rises among particles suspending in a fluid. Particles with high surface charges of the same sign repel each other and do not tend to agglomerate. Thus, high absolute ζ-values (negative or positive) generally indicate that suspensions systems are electrically stable. The ζeta potential of prepared FLG nanofluids was investigated for a month as for DLS measurements, and the obtained data are gathered in Table 1. Samples exhibit ζ-values in the range between −34 and −40 mV, which are (in absolute value) higher than the ±30 mV threshold usually regarded as efficient stability proof for water-based suspensions [9,24]. As can be observed in Table 1, no significant modification of this physicochemical property was detected in the investigated timeframe. These results evidence both the robustness of the exfoliation process and the good stability of nanofluids thus obtained.

### 3.3. Thermophysical Profile

#### 3.3.1. Density and Thermal Expansivity

**Figure 4:**
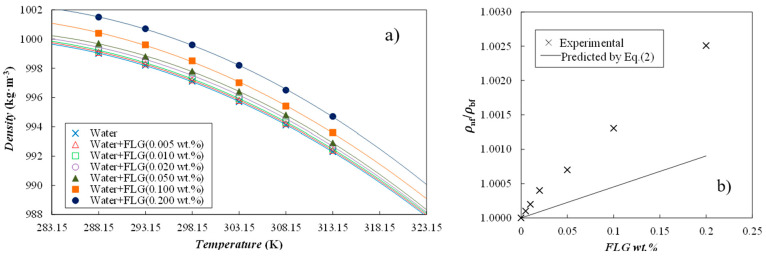
(**a**) Temperature dependence of density for the investigated nanofluid set. (—) Second-order polynomial fittings, Equation (1), and (**b**) relative densities, *ρ*_nf_/*ρ*_bf_, versus FLG content at 303.15 K.

Density was experimentally evaluated at atmospheric pressure and from 288.15 K to 313.15 K, at each 5 K. Obtained values for the base fluid and the six prepared nanofluid concentrations are depicted in Figure 4a. Water data agree well with well-stablished reference [25]. As can be observed in Figure 4b, this thermophysical property increases with FLG loading, with modifications that reach 0.25% at the 0.200 wt.% nanoparticle concentration. Similar changes in density for a 0.25 wt.% content was obtained by Vallejo et al. [26] when investigating functionalized-GNP nanofluids based on a propylene glycol:water mixture at 30:70% mass ratio. Experimental data were correlated with temperature according to the following polynomial fitting:

*ρ*(*T*) = a_0_ + a_1_·T + a_2_·T^2^ (1)

where *ρ* is the density in kg·m^−3^, T is the temperature in K and a_i_ are the fitting parameters (gathered in Table 2, together with standard deviations). Nanofluid *ρ* values at 283.15 K and 323.15 K were determined with Equation (1). From 283.15 to 323.15 K, this property decreases by about 1.2% for both the base fluid and the developed FLG nanofluids. Isobaric thermal expansivity coefficients, α_p_ = −(1/*ρ*)·(∂*ρ*/∂T)_p_, were also calculated from density correlations. At investigated conditions, this thermophysical property rises with increasing temperature from about 2.0·10^−4^ K^−1^ at 293.15 K to 3.0·10^−4^ K^−1^ at 313.15 K. Even if α_p_ values were slightly higher, as in the case of 0.050–0.200 wt.% FLG nanofluids, maximum variations were within 0.06 K^−1^.

A comparison of experimental nanofluid densities to the following mass-averaged equation was also performed:

(2) $$\frac{1}{\rho_{nf}}=\frac{\varphi_{m}}{\rho_{np}}+\frac{\left(1-\varphi_{m}\right)}{\rho_{bf}}$$

where *ρ* is the density and *φ_m_* is the mass fraction of nanoparticles, while *nf*, *np* and *bf* subscripts stand for nanofluid, nanoparticles and base fluid, respectively. In this case, a density of 1820 kg·m^−3^ was considered for the FLG nanopowder [27]. As shown in Figure 4b, experimental density increases are higher than predicted by Equation (2), with deviations that reach 0.16% at the highest content of FLG (0.200 wt.%).

#### 3.3.2. Isobaric Heat Capacity

**Figure 5:**
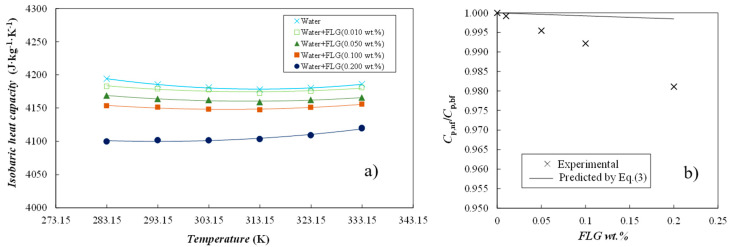
(**a**) Isobaric heat capacities as a function of temperature. (—) Second-order polynomial fittings and (**b**) relative isobaric heat capacities, *Cp_nf_*/*Cp_bf_*, as a function of FLG content at 303.15 K.

Isobaric heat capacities, *Cp*, were investigated for the dry FLG powder, the base fluid (water) and four representative FLG dispersions (viz., 0.010, 0.050, 0.100 and 0.200 wt.%) from 283.15 to 333.15 K. Obtained experimental results are shown in Figure 5a. The *Cp* values measured for the dry FLG powder increase from 899 J·kg^−1^·K^−1^ at 283.15 K to 1106 J·kg^−1^·K^−1^ at 333.15 K. Values are in the range of 700–1600 J·kg^−1^·K^−1^, usually reported for carbon-based materials at similar temperature conditions [28,29,30,31]. Nanofluids exhibit the same temperature trend as water, with a minimum at around 303–313 K [25]. As shown, *Cp* of nanofluids weakly decreases with the increasing FLG content in the dispersion. Thus, in comparison to water, the *Cp* values of 0.010 and 0.200 wt.% concentrations are, on average, 0.15% and 1.9% lower, respectively. Experimental data were confronted to the isobaric heat capacities calculated by means of the Xuan and Roetzel [32] correlation, defined by the following equation:

(3) $${Cp}_{nf}=\varphi_{m}\cdot {Cp}_{np}+\left(1-\varphi_{m}\right)\cdot {Cp}_{bf}$$

in which *φ_m_* is the nanoparticle mass fraction and *Cp_nf_*, *Cp_np_* and *Cp_bf_* are the isobaric heat capacities of the nanofluid, nanoadditive and base fluid, respectively. Figure 5b graphically compares the reductions in this property experimentally obtained and calculated by means of Equation (3). Even if both data sets show the same trend with FLG content, experimental reductions are higher than expected, according to Equation (3). The same observations from Żyła et al. [33] have been made, when investigating ethylene glycol-based nanofluids loaded with various types of nitrides.

#### 3.3.3. Thermal Conductivity

**Figure 6:**
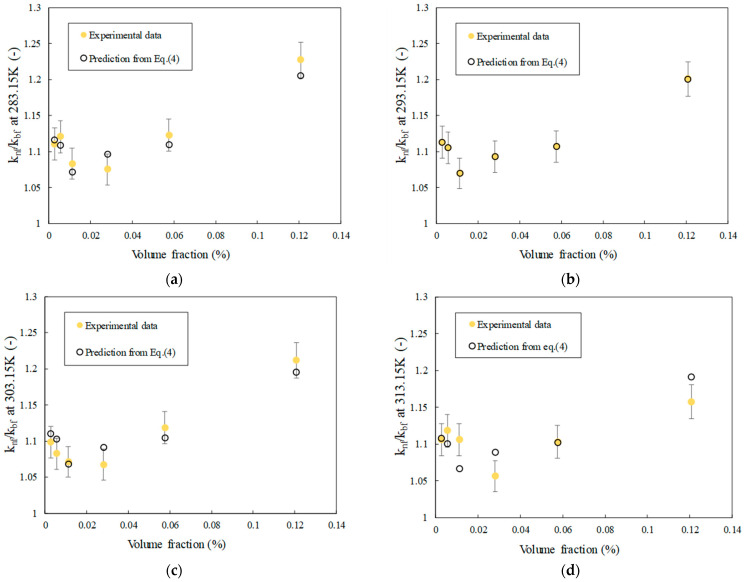
Thermal conductivity ratio of FLG nanofluids as a function of FLG volume fraction at different temperatures (**a**–**d**)—Comparison of experimental data and predictions of Equation (4).

Figure 6a–d show the evolution of thermal conductivity ratio, defined as the thermal conductivity of nanofluids divided by the thermal conductivity of base fluid distilled water (DW) as a function of temperature and FLG volume fraction. As an expectation, the thermal conductivity is enhanced by both FLG content and temperature increase compared to DW. A 20% increase in average is obtained at 0.200% wt. content of graphene for the range of tested temperatures. For comparison purposes, an enhancement of about 12% is achieved at 0.100 wt.% and 313.15 K, that is, higher than 7.9%, reported by Gao et al. [34] for FLG/water nanofluids under the same conditions of concentration and temperature. In interesting ways, this enhancement reaches an average of 10% for only 0.005 wt.% graphene. A closer look at the results also evidences that thermal conductivity tends to decrease for intermediate concentrations in FLG but with reasonable enhancement of about 7% compared to DW. Such a trend for thermal conductivity was yet reported previously for carbon nanotubes and water-based nanofluids [35].

It is now well-established that thermal conductivity of nanofluids depends on several coupled mechanisms and parameters of thermal conductivities of base fluid and nanoparticles, including their content, size, shape, motion and distribution. One relevant theoretical thermal conductivity correlation for graphene-based composites and nanofluids is Chu’s model [8,36]. In order to analyze the thermal conductivity evolution of the FLG nanofluids, this equation is considered here. This model, defined by Equation (4), includes parameters such as the flatness ratio, length, thickness, volume fraction, and interfacial thermal resistance of FLG nanosheets:

(4) $$k_{nf}=k_{bf}\left(\frac{3+\frac{2\eta^{2}\varphi }{\left[k_{bf}\left(\frac{2R_{K}}{L}+13.4\sqrt{t}\right)\right]}}{3-\eta \varphi }\right)$$

where *L* is the average length of graphene, taken here is 5 μm, and *t* is the average thickness of 5 nm, as evidenced previously by TEM characterization. R_K_ is the interfacial thermal resistance, taken at 4.5 × 10^−8^ m^2^·K·W^−1^ at 293.15 K [37], considering the number of layers and that the base fluid is water. Finally, *φ* is the FLG volume fraction expressed by the following relationship, which depends on FLG mass fraction, denoted as *φ_m_*, and the density of the base fluid, *ρ_bf_*, typically water, and FLG nanoparticles, *ρ_np_*, at 293.15 K (reported above in Section 3.3.1). It should be noted that the density of karaya gum was not considered because of the low content used for nanofluid preparation and the value near the density of water.

(5) $$\varphi =\frac{\varphi_{m}\frac{\rho_{bf}}{\rho_{np}}}{\left(1-\varphi_{m}\left(1-\frac{\rho_{bf}}{\rho_{np}}\right)\right)}$$

**Figure 7:**
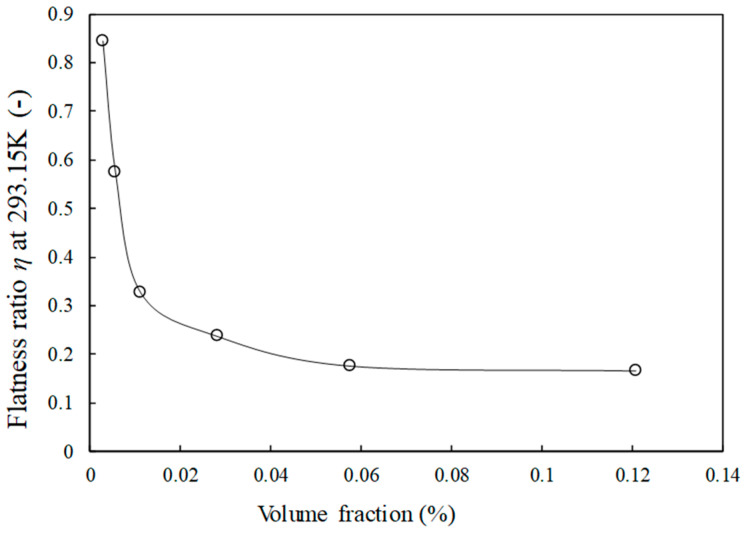
Flatness ratio evolution of FLG in Chu’s Model as a function of FLG volume fraction at 293.15 K (The line is only a guide for the eye to follow).

Consequently, all the parameters from these equations are fixed, except the flatness ratio, *η*, which characterizes the ability of FLG to be bended and wrinkled within the base fluid, and will be adjusted. Hence, Figure 6a–d also presents the results of the analysis performed using the Chu model. These predictive values are then compared to the measured experimental data to evaluate the model’s effectiveness and precision. Consequently, an Average Absolute Deviation (AAD) of 0.035% is obtained at 293.15 K with a flatness ratio evolution as reported in Figure 7. AAD values are 1.3%, 1.4% and 1.9% at 283.15, 303.15 and 313.15 K, respectively. Those results are reasonable when considering the value of the interfacial thermal resistance at 293.15 K only, in the absence of any other relevant reference at lower and higher temperatures.

In Figure 7, a reduction in the flatness ratio (the only adjustable parameter in the model) is observed as the FLG content increases, allowing good prediction of the model compared to the experimental data. This means that with more FLG sheets in the nanofluids, the likelihood of FLG contact and overlap rises, leading to a decrease in the FLG flatness ratio. This occurs as FLGs may undergo deformation, adopting a more curved shape in the fluid [38]. Moreover, larger FLGs are more susceptible to bending and wrinkling due to their size and shape [39]. This observation suggests that a higher number of graphene nanosheets correlates with an increased tendency to bend and wrinkle within the HTF. Such behavior differs from the case of FLG dispersed in fluid composed of water and propylene glycol from non-covalent functionalization, using surfactant in rather large content. In that case, due to the adsorption of many surfactant molecules on the nanosheet surface, a constant flatness ratio was reported [25].

#### 3.3.4. Dynamic Viscosity

**Figure 8:**
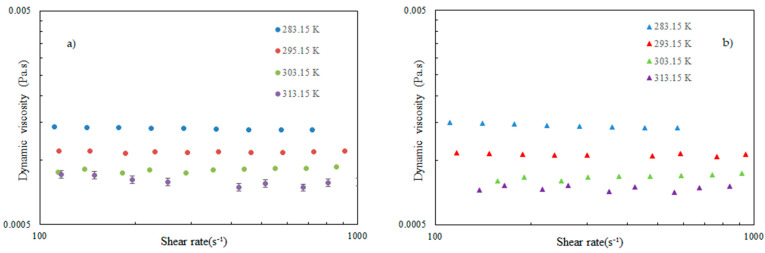
Dynamic viscosity of FLG nanofluids as a function of shear rate for different temperatures and at (**a**) 0.005 wt.% and (**b**) 0.050 wt.% in FLG content.

**Figure 9:**
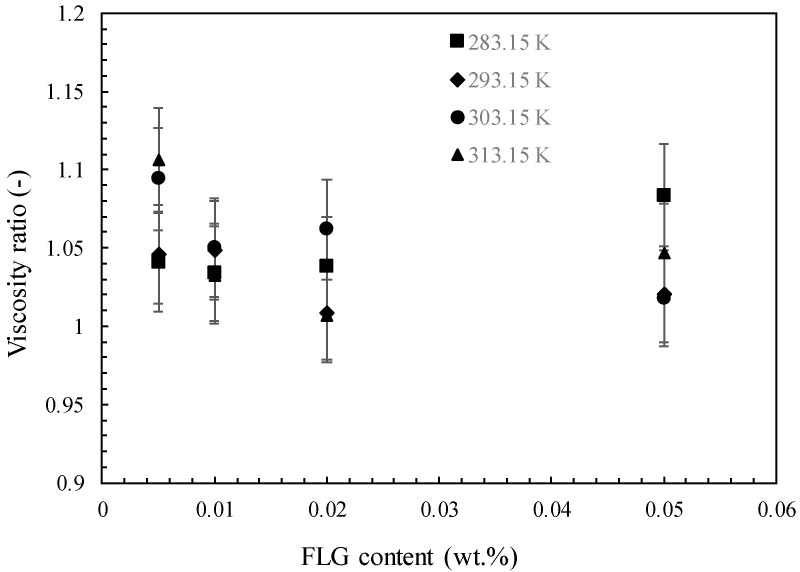
Viscosity ratio of FLG nanofluids as a function of temperature and FLG mass fraction.

Because of the practical implementation of nanofluids in heat transfer systems, it is necessary to investigate how nanoparticle content can impact the dynamic viscosity of nanofluids, which is directly related to pressure drop and power consumption [5]. Consequently, the dynamic viscosity of FLG nanofluids was evaluated at temperatures ranging from 283.15 K to 313.15 K and FLG concentration up to 0.050 wt.%. As illustrated by Figure 8 at 0.005 wt.% and 0.050 wt.%, the nanofluids mainly show a Newtonian nature in the shear rate range investigated within the experimental uncertainty. A viscosity decrease in both temperature increase and FLG content decrease is also evidenced. The temperature-dependent decrease in dynamic viscosity reaches 44%, 48%, 49% and 50% for mass concentrations of 0.005%, 0.010%, 0.020% and 0.050%, respectively. This evolution aligns with previous studies on aqueous nanofluids containing graphene nanoplatelets, as reported by Merhali et al. [40], Vakili et al. [41], Yarmand [42], Vallejo et al. [43] and Alvarado et al. [44]. Also, the presence of FLG induces an enhancement in viscosity compared to distilled water, as highlighted by Figure 9, which reports the viscosity ratio (calculated as the ratio of the viscosity of nanofluids to the viscosity of DW). This figure, as also evidenced by Figure 8, shows that there is no great change in viscosity with FLG content, with average enhancements of about 5%, which are near the experimental uncertainty.

## 4. Conclusions

We report the first comprehensive characterization of the stability and thermophysical properties of Few-Layer Graphene (FLG) nanofluids produced in an industrial context with FLG mass fractions ranging from 0.005% to 0.200%. These nanofluids are developed through a green and smooth mechanical exfoliation process, which ensures FLG production with excellent structural quality and controlled dimensions, as assessed by SEM and HRTEM techniques. Moreover, ζeta potential and DLS analyses also show good stability of these industrial nanofluids. Their thermophysical profile is fully evaluated over a temperature range from 283.15 K to 303.15 K. As expected, the density of the nanofluids slightly increases with FLG content, while the heat capacity weakly decreases. A maximum enhancement of about 20% in thermal conductivity is reported at 0.200 wt.%, while this improvement reaches 10% at only 0.005 wt.%, and the dynamic viscosity remains within a reasonable variation of 5% compared to pure water.

For the majority of the properties, as well as across a range of temperatures and FLG concentrations, the experimental data were found to align well with theoretical correlations. The mass-averaged equation and mixture law provide accurate predictions for density and heat capacity, respectively. Regarding thermal conductivity, the enhancements in this property are demonstrated to be mainly governed by interfacial thermal resistance, average dimensions of FLG and the ability of FLG to be bent and wrinkled within the base fluid. Such behavior depends on FLG content, as illustrated by the model from Chu, here considered.

Finally, based on these properties and stability, industrial graphene-based nanofluids that can be produced in large quantities appear as promising heat transfer fluids in thermal engineering applications, in particular at low graphene content.

## Figures and Tables

**Table 1:** Weekly values of ζeta potentials for 1:100 diluted samples.

FLG Concentration wt.%	ζeta Potential (mV)				
	1st Day	8th Day	16th Day	23rd Day	30th Day
0.005	−35.3 ± 0.3	−36.5 ± 0.5	−38.4 ± 0.6	−36.5 ± 0.7	−34.0 ± 0.2
0.010	−39.2 ± 1.6	−35.3 ± 0.3	−37.9 ± 0.8	−37.1 ± 1.2	−35.5 ± 1.0
0.020	−39.6 ± 0.1	−36.5 ± 0.3	−37.8 ± 0.8	−37.6 ± 1.4	−40.4 ± 2.4
0.050	−38.3 ± 0.4	−39.9 ± 0.2	−38.4 ± 1.1	−38.3 ± 0.9	−36.7 ± 1.3
0.100	−39.7 ± 1.0	−37.2 ± 1.3	−36.1 ± 0.9	−38.8 ± 1.1	−37.3 ± 1.2
0.200	−36.5 ± 0.5	−37.4 ± 1.1	−37.2 ± 0.4	−35.0 ± 0.6	−35.5 ± 0.6

**Table 2:** Fitting parameters (a_0_, a_1_, a_2_) and standard deviations (s).

	FLG Mass Fraction (wt.%)					
	0.005	0.010	0.020	0.050	0.100	0.200
a_0_/(kg·m^−3^)	625.6	625.7	625.8	600.4	544.9	628.5
a_1_/(kg·m^−3^·K^−1^)	2.74	2.74	2.74	2.91	3.29	2.73
−10^3^·a_2_/(kg·m^−3^·K^−2^)	5.00	5.00	5.00	5.29	5.93	5.00
10^2^·s/(kg·m^−3^)	6.1	6.1	9.5	5.1	7.4	5.0

## Data Availability

Data are contained within the article.

## Nomenclature

Abbreviations	
AAD	Average absolute deviation (%)
DLS	Dynamic light scattering
Cp	Isobaric heat capacity, (J·kg^−1^·K^−1^)
DW	Deionized/Distilled water
FLG	Few layer graphene
HTF	Heat transfer fluid
HRTEM	High-resolution transmission electron microscopy
GNP	Graphene nanoplatelets
k	Thermal conductivity (W·m^−1^·K^−1^)
L	Average length of graphene (μm)
MLG	Multilayer/thick graphene
PHP	Pulsed heat pipe
R_K_	Interfacial thermal resistance (m^2^·K·W^−1^)
SEM	Scanning electron microscopy
T	Temperature (K)
t	Graphene thickness (nm)
TEM	Transmission electron microscopy
TMDSC	Temperature-modulated differential scanning calorimetry
Greek letters	
*α_p_*	Isobaric thermal expansivity coefficient (K^−1^)
ζ	Zeta potential (mV)
η	Average flatness ratio of FLG
ρ	Density (kg·m^−3^)
$\varphi_{m}$	Mass fraction
φ	Volume fraction
Subscripts	
bf	Base fluid
nf	Nanofluid
np	Nanoparticles

## References

[1] Tam N.T., Phuong N.V., Khoi P.H., Minh P.N., Afrand M., Van Trinh P., Thang B.H., Zyła G., Estellé P. (2020). Carbon nanomaterial-based nanofluids for direct thermal solar absorption. Nanomaterials.

[2] Novoselov K.S., Geim A.K., Morozov S.V., Jiang D., Katsnelson M.I., Grigorieva I.V., Dubonos S.V., Firsov A.A. (2005). Two-dimensional gas of massless Dirac fermions in graphene. Nature.

[3] Neto A.H.C., Guinea F., Peres N.M.R., Novoselov K.S., Geim A.K. (2009). The electronic properties of graphene. Rev. Mod. Phys..

[4] Parvin N., Kumar V., Joo S.W., Park S.-S., Mandal T.K. (2022). Recent advances in the characterized identification of mono-to-multi-layer graphene and its biomedical applications: A review. Electronics.

[5] Hamze S., Cabaleiro D., Estellé P. (2021). Graphene-based nanofluids: A comprehensive review about rheological behavior and dynamic viscosity. J. Mol. Liq..

[6] Bahiraei M., Heshmatian S. (2019). Graphene family nanofluids: A critical review and future research directions. Energy Convers. Manag..

[7] Pavía M., Alajami K., Estellé P., Desforges A., Vigolo B. (2021). A critical review on thermal conductivity enhancement of graphene-based nanofluids. Adv. Colloid Interface Sci..

[8] Hamze S., Berrada N., Cabaleiro D., Desforges A., Ghanbaja J., Gleize J., Bégin D., Michaux F., Maré T., Vigolo B. (2020). Few-layer graphene-based nanofluids with enhanced thermal conductivity. Nanomaterials.

[9] Le Ba T., Mahian O., Wongwises S., Szilágyi I.M. (2020). Review on the recent progress in the preparation and stability of graphene-based nanofluids. J. Therm. Anal. Calorim..

[10] Sadeghinezhad E., Akhiani A.R., Metselaar H.S.C., Latibari S.T., Mehrali M., Mehrali M. (2020). Parametric study on the thermal performance enhancement of a thermosyphon heat pipe using covalent functionalized graphene nanofluids. Appl. Therm. Eng..

[11] Kumar L.H., Kazi S.N., Masjuki H.H., Zubir M.N.M., Jahan A., Bhinitha C. (2021). Energy, exergy and economic analysis of liquid flat-plate solar collector using green covalent functionalized graphene nanoplatelets. Appl. Therm. Eng..

[12] Balaji T., Selvam C., Lal D.M., Harish S. (2020). Enhanced heat transport behavior of micro channel heat sink with graphene based nanofluids. Int. Commun. Heat Mass Transf..

[13] Xu Y., Xue Y., Qi H., Cai W. (2020). Experimental study on heat transfer performance of pulsating heat pipes with hybrid working fluids. Int. J. Heat Mass Transf..

[14] Alawi O.A., Sidik N.A.C., Kazi S.N., Najafi G. (2019). Graphene nanoplatelets and few-layer graphene studies in thermo-physical properties and particle characterization. J. Therm. Anal. Calorim..

[15] Janowska I., Truong-Phuoc L., Ba H., Pham-Huu C. Nanocomposites Nanomatériau/Système Polymoléculaire Colloïdaux, et Méthodes de Préparation. WO 2018/087484 A1 [Online]. https://patents.google.com/patent/WO2018087484A1/fr.

[16] Cabaleiro D., Gracia-Fernández C., Lugo L. (2014). (Solid+liquid) phase equilibria and heat capacity of (diphenyl ether+biphenyl) mixtures used as thermal energy storage materials. J. Chem. Thermodyn..

[17] Hamze S., Cabaleiro D., Maré T., Vigolo B., Estellé P. (2020). Shear flow behavior and dynamic viscosity of few-layer graphene nanofluids based on propylene glycol-water mixture. J. Mol. Liq..

[18] Osswald S., Havel M., Gogotsi Y. (2007). Monitoring oxidation of multiwalled carbon nanotubes by Raman spectroscopy. J. Raman Spectrosc..

[19] Chizari K., Janowskaa I., Houlléa M., Florea I., Ersenb O., Romeroa T., Bernhardt P., Ledouxa M.J., Pham-Huua C. (2010). Tuning of nitrogen-doped carbon nanotubes as catalyst support for liquid-phase reaction. Appl. Catal. A.

[20] Ferrari A.C., Meyer J.C., Scardaci V., Casiraghi C., Lazzeri M., Mauri F., Piscanec S., Jiang D., Novoselov K.S., Roth S. (2006). Raman spectrum of graphene and graphene layers. Phys. Rev. Lett..

[21] Chakraborty S., Panigrahi P.K. (2020). Stability of nanofluid: A review. Appl. Therm. Eng..

[22] Ilyas S.U., Shamsuddin R., Xiang T.K., Estellé P., Pendyala R. (2023). Rheological profile of graphene-based nanofluids in thermal oil with hybrid additives of carbon nanotubes and nanofibers. J. Mol. Liq..

[23] Fedele L., Colla L., Bobbo S., Barison S., Agresti F. (2011). Experimental stability analysis of different water-based nanofluids. Nanoscale Res. Lett..

[24] Ilyas S.U., Pendyala R. (2014). Preparation sedimentation and agglomeration of nanofluids. Chem. Eng. Technol..

[25] Lemmon E.W., Huber M.L., McLinden M.O. (2007). NIST reference fluid thermodynamic and transport properties database. NIST 23, (REFPROP).

[26] Vallejo J.P., Pérez-Tavernier J., Cabaleiro D., Fernández-Seara J., Lugo L. (2018). Potential heat transfer enhancement of functionalized graphene nanoplatelet dispersions in a propylene glycol-water mixture. Thermophysical profile. J. Chem. Thermodyn..

[27] Hamze S., Cabaleiro D., Bégin D., Desforges A., Maré T., Vigolo B., Lugo L., Estellé P. (2020). Volumetric Properties and Surface Tension of few-layer graphene nanofluids based on a commercial heat transfer fluid. Energies.

[28] Picard S., Burns D.T., Roger P. (2007). Determination of the specific heat capacity of a graphite sample using absolute and differential methods. Metrologia.

[29] Picard S., Burns D.T., Roger P. (2006). Measurement of the Specific Heat Capacity of Graphite.

[30] Marcos M.A., Cabaleiro D., Guimarey M.J.G., Comuñas M.J.P., Fedele L., Fernández J., Lugo L. (2017). PEG 400-based phase change materials nano-enhanced with functionalized graphene nanoplatelets. Nanomaterials.

[31] Pop E., Varshney V., Roy A.K. (2012). Thermal properties of graphene: Fundamentals and applications. MRS Bull..

[32] Xuan Y., Roetzel W. (2000). Conceptions for heat transfer correlation of nanofluids. Int. J. Heat Mass Transf..

[33] Żyła G., Vallejo J.P., Lugo L. (2018). Isobaric heat capacity and density of ethylene glycol based nanofluids containing various nitride nanoparticle types: An experimental study. J. Mol. Liq..

[34] Gao Y., Wang H., Sasmito A.P., Mujumdar A.S. (2018). Measurement and modeling of thermal conductivity of graphene nanoplatelet water and ethylene glycol base nanofluids. Int. J. Heat Mass Transf..

[35] Maré T., Halelfadl S., Van Vaerenbergh S., Estellé P. (2015). Unexpected sharp peak in thermal conductivity of carbon nanotubes water-based nanofluids. Int. Commun. Heat Mass Transf..

[36] Chu K., Li W., Tang F. (2013). Flatness-dependent thermal conductivity of graphene-based composites. Phys. Lett. A.

[37] Alexeev D., Chen J., Walther J.H., Giapis K.P., Angelikopoulos P., Koumoutsakos P. (2015). Kapitza resistance between few-layer graphene and water: Liquid layering effects. Nano Lett..

[38] Fu S., Sun Z., Huang P., Li Y., Hu N. (2019). Some basic aspects of polymer nanocomposites: A critical review. Nano Mater. Sci..

[39] Androulidakis C., Koukaras E.N., Rahova J., Sampathkumar K., Parthenios J., Papagelis K., Frank O., Galiotis C. (2017). Wrinkled few-layer graphene as highly efficient load bearer. ACS Appl. Mater. Interfaces.

[40] Sadeghinezhad E., Mehrali M., Rosen M.A., Akhiani A.R., Latibari S.T., Mehrali M., Metselaar H.S.C. (2016). Experimental investigation of the effect of graphene nanofluids on heat pipe thermal performance. Appl. Therm. Eng..

[41] Vakili M., Khosrojerdi S., Aghajannezhad P., Yahyaei M. (2017). A hybrid artificial neural network-genetic algorithm modeling approach for viscosity estimation of graphene nanoplatelets nanofluid using experimental data. Int. Commun. Heat Mass Transf..

[42] Yarmand H., Gharehkhani S., Shirazi S.F.S., Amiri A., Alehashem M.S., Dahari M., Kazi S. (2016). Experimental investigation of thermo-physical properties, convective heat transfer and pressure drop of functionalized graphene nanoplatelets aqueous nanofluid in a square heated pipe. Energy Convers. Manag..

[43] Vallejo J.P., Gómez-Barreiro S., Cabaleiro D., Gracia-Fernández C., Fernández-Seara J., Lugo L. (2018). Flow behaviour of suspensions of functionalized graphene nanoplatelets in propylene glycol–water mixtures. Int. Commun. Heat Mass Transf..

[44] Wang Y., Al-Saaidi H.A.I., Kong M., Alvarado J.L. (2018). Thermophysical performance of graphene based aqueous nanofluids. Int. J. Heat Mass Transf..

